# Nested autoinhibitory feedbacks alter the resistance of homeostatic adaptive biochemical networks

**DOI:** 10.1098/rsif.2013.0971

**Published:** 2014-02-06

**Authors:** Jörg Schaber, Anastasiya Lapytsko, Dietrich Flockerzi

**Affiliations:** 1 Institute for Experimental Internal Medicine, Medical Faculty, Otto von Guericke University, Magdeburg, Germany; 2 Max Planck Institute for Dynamics of Complex Technical Systems, Magdeburg, Germany

**Keywords:** negative feedback control, Hopf bifurcation, high osmolarity glycerol, p53

## Abstract

Negative feedback control is a ubiquitous feature of biochemical systems, as is time delay between a signal and its response. Negative feedback in conjunction with time delay can lead to oscillations. In a cellular context, it might be beneficial to mitigate oscillatory behaviour to avoid recurring stress situations. This can be achieved by increasing the distance between the parameters of the system and certain thresholds, beyond which oscillations occur. This distance has been termed resistance. Here, we prove that in a generic three-dimensional negative feedback system the resistance of the system is modified by nested autoinhibitory feedbacks. Our system features negative feedbacks through both input-inhibition as well as output-activation, a signalling component with mass conservation and perfect adaptation. We show that these features render the system applicable to biological data, exemplified by the high osmolarity glycerol system in yeast and the mammalian p53 system. Output-activation is better supported by data than input-inhibition and also shows distinguished properties with respect to the system's stimulus. Our general approach might be useful in designing synthetic systems in which oscillations can be tuned by synthetic autoinhibitory feedbacks.

## 1. Introduction

Negative feedback control is a fundamental and a ubiquitous feature of biochemical systems [1–6] and can mediate adaptation [7–10], stabilize the abundance of biochemical species [4,11,12], induce oscillations [3,5,13–16] and accelerate response times [11,17]. In fact, negative feedbacks have been observed in a wealth of biological systems ranging from mammalian cell cycle [13,18] to bacterial adaptation [8,19] and stress response in mammals [20] and yeast [21,22]. Another ubiquitous principle of biochemical systems is time delay between a signal and its response, which can, for example, be caused by the time needed to transcribe and translate biochemical information into cellular compounds. It is a long-standing theoretical result that negative feedbacks in conjunction with time delay can lead to stable oscillations [15,16,23]. Oscillatory behaviour brought about by delayed negative feedbacks has been observed and analysed in a range of biological systems, for example, the mammalian p53 system [24–26], and the NF-κB system [27–29]. Both the p53 as well as the NF-κB system mediate adaptation to external stimuli and stress, such as, for example, DNA damage. It is comprehensible that it might be beneficial to mitigate oscillatory behaviour during adaptation in order to avoid recurring stress situations. This can be achieved by moving the steady state of the system far away from certain thresholds, beyond which oscillations occur. The distance between such thresholds and the parameters of the system has been termed resilience and/or resistance. The larger the resistance of a system, the better perturbations in external or internal conditions, i.e. the systems parameters, can be absorbed, which otherwise would trigger a change in stability properties of the system [30,31]. There exist several definitions of resistance in the literature [31]. Here, based on [31], we will refer to ‘resistance’ as the system's response to perturbations of parameter values. As a quantitative measure of resistance, we use the Euclidian distance of the parameter vector to a critical threshold, beyond which stability properties of the system change. The larger this distance, the more resistant is the system.

In a recent study, using a parametrized mathematical model, evidence was presented that during osmo-adaptation in yeast, which is largely mediated by a delayed negative integral feedback, the potential of oscillatory behaviour is reduced by introducing nested direct negative feedbacks [21]. Thus, there is evidence that in a concrete biological system nested negative feedbacks can increase the resistance of a biochemical network.

Here, using a generic three-dimensional model for integral negative feedback control of a biochemical network, we explore whether coupling autoinhibitory and delayed negative feedbacks might be a general cellular mechanism to increase resistance of a system. Our system has several distinguished features which generalize and extend former studies [15,16,32]:

— our models include components, which resemble post-translational modification of proteins conserving the total protein abundance (mass conservation), rendering the system more general and, at the same time, more realistic, especially with respect to signalling cascades;
— our models use integral feedback properties, i.e. some state variables of the system robustly track their desired values independent from the input [8], which also renders the systems more applicable to realistic situations, as shown below; and
— in our models, the delayed negative feedback may operate through both by inhibiting sensor inflow, like in [15], and by activating sensor outflow, like in [9]. This has been termed input and output control [10]. Here, we refer to these two control types as input-inhibition and output-activation, respectively. The type of used delayed negative feedback has important implications with respect to what actually stimulates the system. We show that an output-activation feedback mechanism is better supported by a range of data.

We prove that, in our systems, stable limit cycle oscillations can occur owing to a Hopf bifurcation. Further, we prove that the parameter region, where oscillations occur, can be reduced by introducing autoinhibitory feedbacks. Thus, by nesting autoinhibitory negative feedbacks into delayed negative feedbacks the structural stability of the system can be altered. This is true, in general, for input-inhibition systems. However, there exist certain limitations for this effect in output-activation systems. We also provide computational evidence that the sensitivity of the steady state with respect to parameter perturbations is decreased in system with nested autoinhibitory feedback. We apply our generic model to the high osmolarity glycerol (HOG) system in yeast, and the mammalian p53 system demonstrating the applicability of our general framework to concrete situations. We propose that this simple framework can be used to design synthetic systems in which oscillatory behaviour can be tuned by nesting direct, possibly autoinhibitory, and delayed negative feedbacks.

## 2. Results

### 2.1. The model

**Figure 1.**
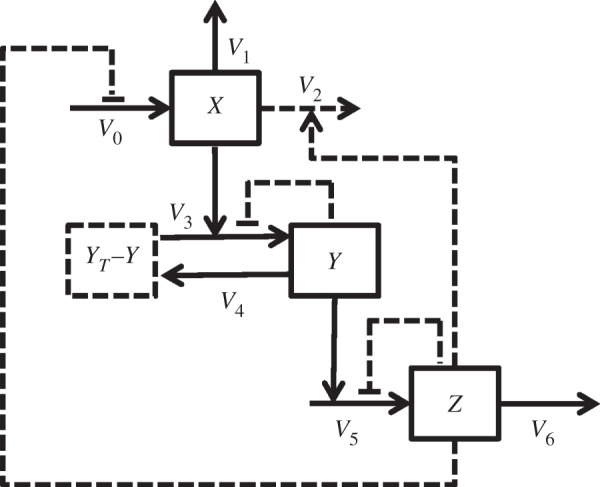
Wiring scheme of the generic integral feedback the model in (2.1). Dashed components indicate model alternatives, which were considered for concrete applications and the examples in the electronic supplementary material. Alternative kinetics are not indicated. Reaction numbers correspond to parameter numbers in (2.1). Specific wiring schemes can be found in the electronic supplementary material along with the respective examples.

In the following, we consider the three-dimensional system2.1
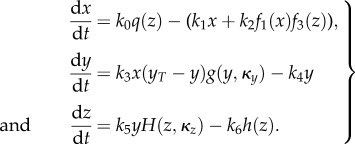
with positive parameters *k*_0_, *k*_1_, *k*_2_, *k*_3_, *k*_4_, *k*_5_, *k*_6_, *y_T_*, *κ*_*y*_, *κ*_*z*_ and non-negative initial conditions *x*_0_, *y*_0_ and *z*_0_. External perturbations are simulated by modifying the value of *k*_0_ which otherwise mimics a basal stimulation of the system. This system can be even more generalized by replacing all linear functions *x* and *y* by smooth strictly increasing functions (see the electronic supplementary material). Figure 1 displays a wiring diagram of the system (2.1) also indicating different alternative model formulations that were tested in the application described below.

In general, *x*(*t*) can be considered as a cellular sensor, which reacts to an external stimulus *k*_0_. The component *y*(*t*) mimics a signal transduction, which relays the input signal coming from the sensor *x*(*t*) to the response component *z*(*t*), which in turn negatively feeds back into the sensor *x*(*t*) via *f*_3_(*z*) or via *q*(*z*). We assume *f*_1_,*f*_3_, and *h* to be strictly increasing functions on 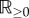 vanishing at 0. The function *q*(*z*) is a smooth, positive and strictly decreasing function with *q*(0) = 1. The functions *f*_3_(*z*) and *q*(*z*) represent the overall feedback in the system, respectively. In the examples below, we consider them to be mutually exclusive and refer to a feedback through *q*(*z*) as input-inhibition, and a feedback through *f*_3_(*z*) as output-activation. The functions *g*(*y*, *κ*_*y*_) and *H*(*z*, *κ*_*z*_) are smooth, positive and decreasing in both arguments with 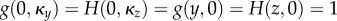. The functions *g*(*y*, *κ*_*y*_) and *H*(*z*, *κ*_*z*_) may represent autoinhibitory feedbacks, where we later consider the additional parameters *κ*_*y*_ and *κ*_*z*_, which, among other parameters, shape the form of these functions. We call them autoinhibitory, because the inhibition of a component depends only on itself and not on other system variables.

The term (*y_T_* − *y*) leads to a model with an *a priori* bound *y* for the second component in case its initial value *y*(0) is in [0, *y_T_*]. This term is obtained by reducing a four-dimensional 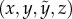 system with 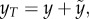 mimicking a reversible post-translational modification, such as, for example, phosphorylation, of a protein *y* that does not affect the total protein abundance *y_T_* (mass conservation).

Note that the non-negative orthant 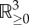 is positive invariant for all models and, therefore, all models are biologically sound in the sense that no negative values for the components can occur. For further details, please refer to the electronic supplementary material.

Taken together, we analyse a generic model that comprises a range of special cases that have been addressed in the literature, for example, the Goodwin-type models [15,16,32], but also addresses models that have not been thoroughly analysed yet, especially the output-activation models, which will be shown to be especially relevant in concrete situations.

### 2.2. Integral feedback property

Integral feedback control is an engineering strategy that is supposed to ensure that the output of a system always adapts to its desired value independent of noise and of perturbations of the system parameters [8]. For two-dimensional systems, it has been reported that the kinetic nature of *h*(*z*) is important in this respect; for example, mass action kinetics for *h*(*z*) is not sufficient to obtain perfect adaptation for *y*(*t*) [9,10]. For convenience of the mathematical analysis, we approximate a zero-order *h*(*z*) by a smooth function 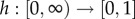 with *h*(0) = 0, and *h*(*z*) = 1 for *z* ≥ *a* > 0 and that is strictly increasing on (0, *a*) and require that an equilibrium 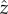 exists with 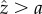. Thus, 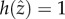 and, for 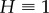, the equilibrium of the second component
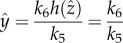
is constant and independent of the input signal in this limiting case.

Taken together, the system (2.1) approximates a perfect adaptor for zero-order *h*(*z*) with respect to *y*(*t*). Note that the above approximation is only a theoretical one ensuring that the solution stays in the positive orthant 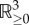, unlike the approximation *K_z_* = 0 for Michaelis–Menten type *h*(*z*) = *z*/(*K_z_* + *z*) for which negative solutions can occur. For concrete situations, it suffices to assume a sufficiently small *K_z_* for the Michaelis–Menten type *h*(*z*) implying 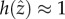.

### 2.3. The output-activation system is stimulated by the difference between the internal and external state

**Figure 2.**
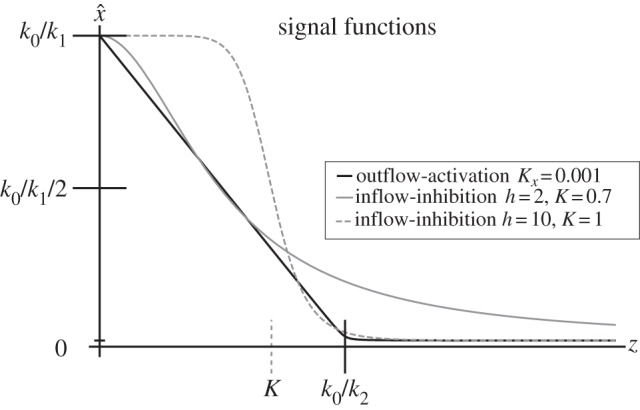
Signal functions, i.e. quasi-equilibrium sensor values 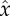 as a function of the response variable *z* for the system (2.1) with either 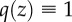 and 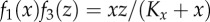
(approximated by (2.2); black curve) or *k*_2_ = 0 and 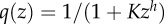 (2.4) (grey curves).

Zero-order kinetics for *f*_1_(*x*) has important implications with respect to what is actually sensed and integrated as error function by the system. Let us assume 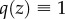, a linear feedback function *f*_3_(*z*) = *z*, and that the sensor *x*(*t*) is in quasi-equilibrium with respect to the response variable *z*. Further, we approximate a zero-order *f*_1_(*x*), for example, 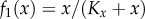, by a smooth function 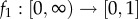 with *f*_1_(0) = 0, and *f*_1_(*x*) = 1 for 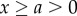 and that is strictly increasing on (0, *a*) and require that at the equilibrium 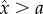. Thus, 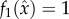 and2.2
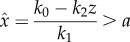
is a linear function of *z*. Thus, for zero-order 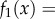 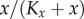 and linear output-activation, the system (2.1) is stimulated by the positive difference between the external stimulus *k*_0_ and the scaled response variable *z*, i.e. the internal state. As above, this approximation is introduced for convenience of the theoretical analysis only. In real situations, it suffices to assume a sufficiently small *K_x_* as in figure 2.

In the case of mass action kinetics for *f*_1_(*x*),2.3
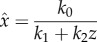


is related to the ratio between the external stimulus *k*_0_ and the response variable *z*. A similar form for 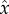 as in (2.3) is obtained in a model with input-inhibition, i.e. *k*_2_ = 0 and
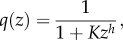
which was assumed in the classical model of Goodwin [15,16,32]. Here, we obtain2.4
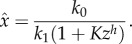
In figure 2, we display quasi-equilibrium sensor values 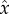 as a function of the response variable *z* for the system (2.1) with either a Hill-type input-inhibition term as in [15] and *k*_2_ = 0, or a zero-order output-activation term with linear feedback in *z*, i.e. 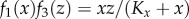 with 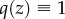, respectively.

Note that in a system where the feedback is mediated through output-activation by a linear feedback with zero-order degradation, i.e. 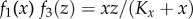 and 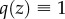, the response threshold *k*_0_/*k*_2_ is well defined for small *K_x_* and can directly be tuned through *k*_2_ (figure 2). Similarly, for systems with Hill-type input-inhibition, i.e. *k*_2_ = 0 and 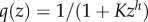, the response threshold is well defined for large Hill factors *h* (grey dashed line in figure 2), but can only indirectly be tuned through the half-saturation constant *K*. In addition, the better the threshold is defined by large Hill factors *h*, the sooner the sensor activation saturates for decreasing *z*, whereas in a system with output-activation, sensor activation is linear below the activation threshold *k*_0_/*k*_2_. Conversely, when in a system with Hill-type input-inhibition, the sensor approximates a linear function in *z* (grey solid line in figure 2), the response threshold is poorly defined.

Taken together, the type (input-inhibition or output-activation) and kinetic nature (mass action or saturating) of the overall negative feedback determines the signal, which stimulates the system. In the case of zero-order output-activation, the system is linearly stimulated by the difference between internal and external conditions; otherwise, the stimulus is nonlinear and may even be step-like. In the examples below, we show that the data clearly support output-activation models rather than support nonlinear Goodwin-type input-inhibition models.

### 2.4. The Hopf bifurcation

In the system (2.1), stable limit cycles can occur due to a Hopf bifurcation. Shortly, any steady state 
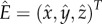
 of the system (2.1) is given by2.5
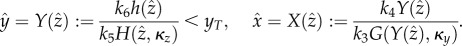


We define *G*(*y*, *κ*_*y*_) = (*y_t_* − *y*)*g*(*y*, *κ*_*y*_) and the parameter value2.6
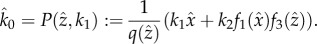


The Jacobian at the equilibrium 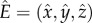 is of the form



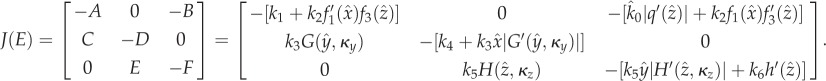


and has the characteristic Hurwitz-polynomial 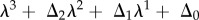, with positive
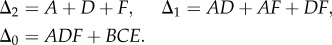


So, any real eigenvalue of *J* is negative. The necessary and sufficient condition for a single pair 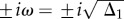 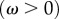 of pure imaginary eigenvalues is 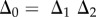, i.e.2.7
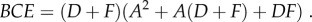
evaluated at 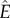. With
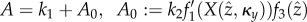



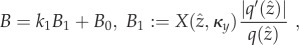



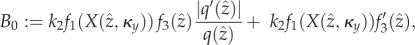


we consider (2.7) as an equation of the parameters *k*_1_ and 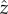 that is to be solved in the form 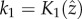. The curve 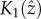, given by the unique positive solution 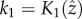 of the quadratic equation, derived from (2.7),2.8
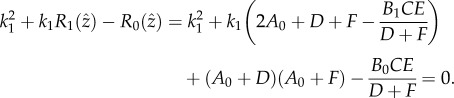
evaluated at 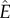, indicates possible Hopf-bifurcation points in the 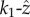-plane. The necessary and sufficient condition of the existence for a positive 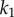-solution of (2.8) is 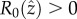, i.e.2.9
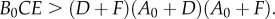


Taken together, having chosen the equilibrium component 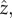 we define 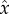 and 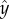 according to (2.5), solve (2.8) for 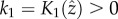, provided (2.9), and set 
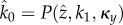
 according to (2.6). Then, the possible Hopf-bifurcation point is given by 
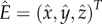
 for the critical parameters *k*_1_ and 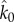.

In this case, the transversality condition for a Hopf bifurcation can be generically fulfilled (see the electronic supplementary material, where also further details of the proof are supplied). Thus, the system (2.1) can show stable oscillations owing to a Hopf bifurcation.

A Hopf bifurcation can also occur for a more general class of systems, where all linear functions *x* and *y* in the system (2.1) are replaced by smooth strictly increasing functions and with or without the term (*y_T_* − *y*) (see the electronic supplementary material).

### Autoinhibition decreases the oscillatory 
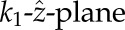


2.5.

In the electronic supplementary material, we prove that the 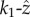-plane permissive for oscillations decreases with increasing autoinhibition either through *g*(*y*, *κ*_*y*_) or *H*(*z*, *κ*_*z*_), i.e. for the curve 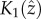 that divides the 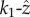-plane into regions with and without stable oscillations it holds2.10
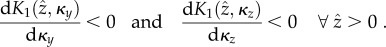


Again, there is a notable difference between models with input-inhibition, i.e. *k*_2_ = 0, and output-activation, i.e. 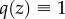.

For models with input-inhibition relation, (2.10) is always true. This has been shown before for the classical Goodwin-type models with Hill-type *q*, i.e. 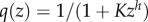 [32]. However, opposed to these classical models, where a high Hill coefficient (cooperativity) of *h* ≥ 8 was necessary to obtain oscillations, this is not necessary in our framework (2.1) (see the electronic supplementary material, figures S4 and S7).

For models with output-activation condition (2.10) only applies, if 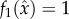, i.e. if at the equilibrium *f*_1_ is of zero-order such that 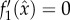. This situation can be approximated, e.g. by low *K_x_* values for 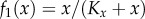. Note that this was also a prerequisite for the quasi-steady state 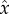 to be a linear function of *z* as in (2.2). However, to reduce the parameter region for oscillations for the output-activation system, 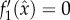 is sufficient, but not necessary (for examples, refer to the electronic supplementary material).

**Figure 3.**
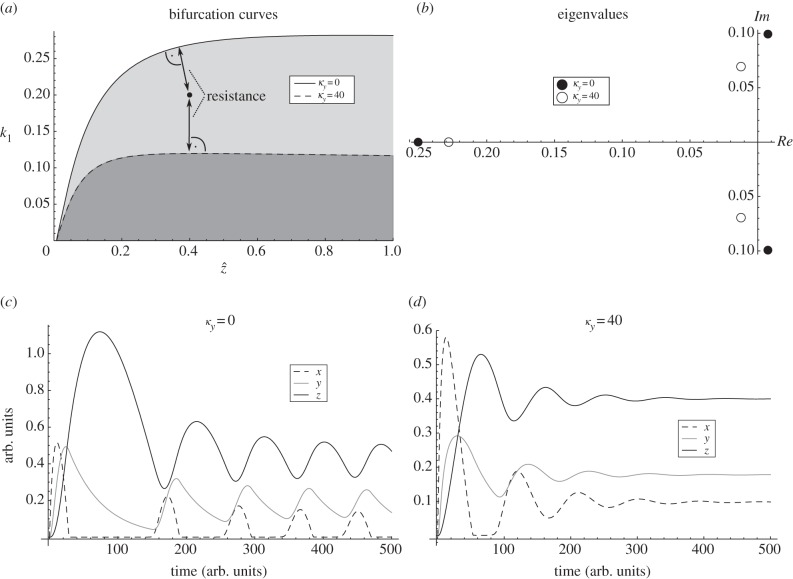
Bifurcation curves, eigenvalues and dynamics for the system (2.1) with 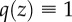, 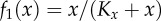, *f*_3_(*z*) = *z*, 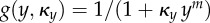 and 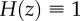, *h*(*z*) = *z*/(*K_z_* + *z*) . (*a*) Bifurcation curves in 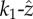-plane for *κ*_*y*_ = 0 and *κ*_*y*_ = 40, respectively. The distance between the parameters of the system, in this case 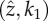, and the bifurcation threshold, i.e. 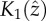, can be interpreted as a measure for resistance. (*b*) Eigenvalues of the Jacobian of the system (2.1) for *κ*_*y*_ = 0 and *κ*_*y*_ = 40, respectively. (*c*) Simulations for 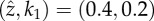 and *κ*_*y*_ = 0. (*d*) Simulations for (*z*_0_, *k*_1_) = (0.4, 0.2) and *κ*_*y*_ = 40. Other parameters: *k*_2_ = 0.3, *k*_3_ = *k*_5_ = 0.1, *k*_4_ = *k*_6_ = 0.02, *K_x_* = 0.0001, *K_z_* = 0.05, *m* = 2.

Taken together, we provide formal proof that the structural stability of the system (2.1) can be altered by introducing autoinhibitory feedbacks. Moreover, the distance between the bifurcation threshold and a given 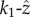 pair can be modulated by introducing autoinhibitory negative feedbacks. Thus, nested autoinhibitory feedbacks can modulate the resistance of the system (2.1). Figure 3 illustrates these theoretical results for an output-activation system. For other examples, please refer to the electronic supplementary material, figures S2–S7.

**Figure 4.**
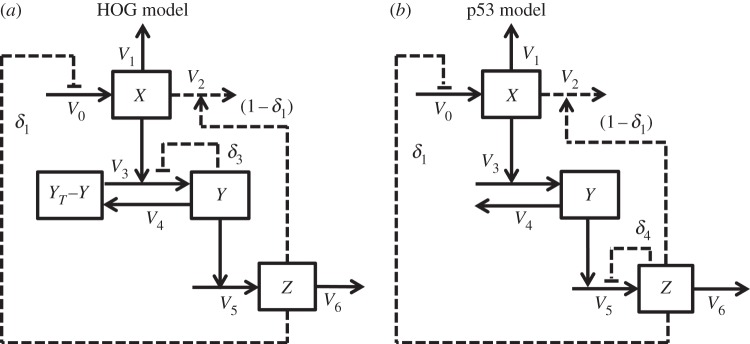
Subset of model alternatives from the system (2.1) (figure 1) tested for the HOG (2.11) and the p53 system (2.12). (*a*) Wiring schemes of the HOG system. Dashed components indicate the considered model alternatives that correspond to the indicated *δ*-values in (2.11). (*b*) Wiring schemes of the p53 system. Dashed components indicate the considered model alternatives that correspond to the indicated *δ*-values in (2.12). Components names are adapted for the concrete system. Alternative kinetics are not indicated. Reaction numbers correspond to parameter numbers in the system (2.1).

In figure 3*a*, we show bifurcation curves in the 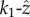-plane, for different values of *κ*_*y*_, i.e. with (*κ*_*y*_ = 40) or without (*κ*_*y*_ = 0) autoinhibitory feedback for a concrete system. The area below the curve, where oscillations occur, is reduced with *κ*_*y*_ increasing. The larger *κ*_*y*_, i.e. the stronger the autoinhibitory feedback, the smaller the area below the curve. The dot in figure 3*a* indicates a concrete pair of 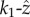, to which the computed eigenvalues in figure 3*b* and dynamics in figure 2*c,d* correspond. The distance between a point in the 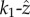 and the bifurcation curve 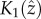 can be interpreted as a measure for resistance. Note that a change in 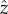 can also be interpreted as a change in parameter 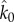, because there is a 1 : 1 relationship between 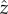 and 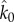 (see (2.6)). Without autoinhibitory feedback (*κ*_*y*_ = 0), the bifurcation parameters are below the bifurcation curve and, consequently, we have a single pair of complex eigenvalues with positive real parts (black dots in figure 3*b*) corresponding to stable oscillations (figure 3*c*). With autoinhibitory feedback (*κ*_*y*_ = 40), the bifurcation parameters are above the bifurcation curve and, consequently, all real parts of the eigenvalues are negative (circles in figure 4*b*), and the system tends to a stable equilibrium (figure 3*d*).

For convenience, we conducted the theoretical analysis by parametrizing the system with respect to the steady state in 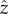 and considered *k*_1_ as the bifurcation parameter. However, as illustrated by the computational analysis of the HOG system and the p53 system below, all tested parameters in the system may be taken as bifurcation parameters (figures 4 and 7; electronic supplementary material, S9 and S10). Accordingly, the introduction of autoinhibitory feedbacks reduces the region for oscillations for those parameters as well.

### 2.6. Application to the high osmolarity glycerol system

The HOG system in yeast mediates adaptation to a hyper-osmotic shock and is one of the best-studied eukaryotic signalling pathways [33]. Several mathematical models of different complexity have been developed for this system [21,22,34,35]. In short, the signal that triggers response, and adaptation is supposedly related to volume [36,37], which, in turn, is proportional to the difference between internal and external osmotic pressure [38]. The signal coming from the membrane is transduced via a stress-activated protein kinase (SAPK) cascade, which culminates in the activation the SAPK Hog1. Hog1 translocates to the nucleus-activating transcription factors that lead to the upregulation of glycerol production, which, in turn, increases the intracellular osmolarity and turgor, thereby mediating adaptation. There is evidence that in this system oscillatory behaviour might indeed be avoided by nested negative feedbacks [21]. Therefore, we tested whether our general framework is supported by available data, and, whether the data support autoinhibitory feedbacks. We fitted different candidate models representing different hypothesis about the underlying biochemical mechanisms and ranked them according to the Akaike information criterion (AIC). The candidate models were specified versions of our general framework (2.1) (figure 4*a*):2.11
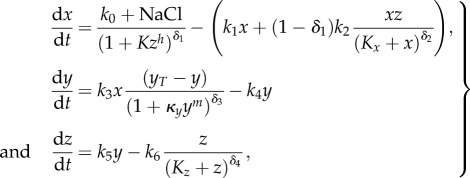
where 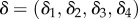 and 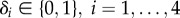 indicate model alternatives. Here, *x* represents a putative sensor of volume change or the difference between internal and external water potentials, i.e. *z* and *k*_0_ + NaCl, respectively. The component *y* represents the adaptive phosphorylated Hog1 and *z* represents the integrator glycerol (figure 4*a*). Specifically, we tested the kind of delayed feedback, i.e. input-inhibition (*δ*_1_ = 1) or output-activation (*δ*_1_ = 0), the existence of autoinhibition in the signalling component *y* (*δ*_3_) and two different kinetics for *f*_1_(*x*) and *h*(*z*), respectively, i.e. mass action (*δ*_2_, *δ*_4_ = 0) or Michaelis–Menten kinetics (*δ*_2_, *δ*_4_ = 1). The combination of all these model alternatives yielded 12 different models. Here, we assumed *f*_3_(*z*) = *z* and *H*(*z*,*κ*_*z*_) ≡ 1. For parameters of the best-ranked model, please refer to the electronic supplementary material, table S2. For more details on the model and parameter estimation, please refer to the Methods section and the electronic supplementary material. A COPASI implementation of the best-ranked model together with the data and an systems biology mark-up language (SBML) [39] version is also provided in the electronic supplementary material. The results of fitting and ranking are displayed in table 1.

**Table 1.** HOG candidate models. *n*, number of data points; *k*, number of parameters; SSR, weighted sum of squared residuals; AIC*c*, Akaike information criterion corrected for small sample size; AIC*w*, Akaike weights.

rank	model no.	*δ*	*n*	*k*	SSR	AIC*c*	AIC*w*
1	6	(0,1,1,0)	67	8	7.5	61.5	0.8
2	8	(0,1,1,1)	67	9	7.5	64.4	0.2
3	2	(0,1,0,0)	67	6	15.3	104.6	0
4	4	(0,1,0,0)	67	7	15.3	107.2	0
5	11	(1,0,1,0)	67	8	16.3	113.9	0
6	9	(1,0,0,0)	67	6	44.3	175.9	0
7	10	(1,0,0,1)	67	7	44.7	179.0	0
8	12	(1,0,1,1)	67	9	44.7	184.2	0
9	1	(0,0,0,0)	67	5	52.0	184.1	0
10	3	(0,0,0,1)	67	6	53.2	188.1	0
11	5	(0,0,1,0)	67	7	52.0	189.1	0
12	7	(0,0,1,1)	67	8	53.2	193.2	0

**Figure 5.**
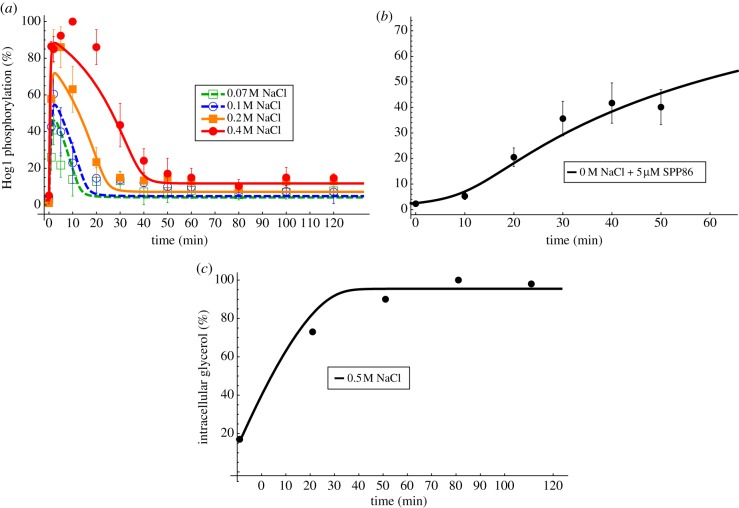
HOG system. Data and fit for the best approximating model no. 6, *δ* = (0, 1, 1, 0). (*a*) Measured (symbols, mean ± s.d. (*n* ≥ 3) from [17]) and simulated (lines) phosphorylated Hog1 (*y*) for different osmotic stress conditions. (*b*) Measured (symbols) and simulated (lines) phosphorylated Hog1 (*y*) in experiments without osmotic shock and where Hog1 kinase activity was inhibited by the kinase inhibitor SPP86. (*c*) Measured (symbols) and simulated (lines) glycerol (*z*) upon an osmotic shock of 0.5 M NaCl. For the source of the experimental data, please refer to section Materials and methods. (Online version in colour.)

The model with zero-order kinetics in *f*_1_(*x*), autoinhibition in *y* and mass action kinetics in *h*(*z*) is ranked best (model no. 6, *δ* = (0, 1, 1, 0)). This corresponds to a model, which senses the difference between external and internal osmolarity and shows no perfect adaptation. This model is closely followed by the same model, but with perfect adaptation (model no. 8, *δ* = (0, 1, 1, 1)). The best model without autoinhibition in *y* is on the third place (model no. 2, *δ* = (0, 1, 0, 0)). The best two models are able to recapitulate Hog1 phosphorylation and intracellular glycerol data for a range of different conditions (figure 5). Model no. 2 can also well recapitulate Hog1 osmotic stress and glycerol data, but cannot recapitulate well the Hog1 inhibition experiment (see electronic supplementary material, figure S8). The models with an input-inhibition did not give a good approximation to the data compared with the best-ranking models (table 1).

**Figure 6.**
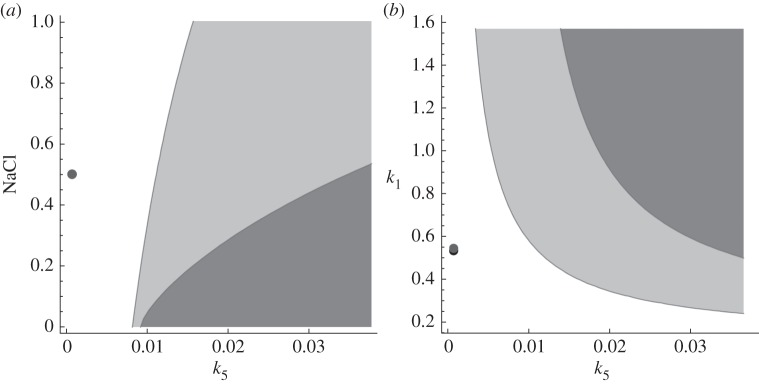
Stability region plots for the HOG system. (*a*,*b*) Shows stability regions for different parameter combinations. In (*a*,*b*), dark grey and light grey indicate regions with unstable steady state, i.e. where oscillations occur, for the system with (model no. 6) and without autoinhibitory feedback (model no. 2), respectively. Black and dark grey dots indicate the fitted parameters for the system with (model no. 6) and without autoinhibitory feedback (model no. 2), respectively. The boundary between white and shaded regions indicates bifurcation lines in the respective two-dimensional parameter space. For more stability region plots for different parameter combinations, please refer to the electronic supplementary material.

Apparently, both the system with and without autoinhibitory feedback can show adaptive behaviour. Analysing the stability of the steady state as a function of the parameters, it becomes obvious that the parameter region, where oscillations occur, is much more distant from the actually fitted parameters for the model with (model no. 6) than for the model without autoinhibition (model no. 2). Thus, the resistance of the adaptation is increased in the system including the autoinhibitory feedback. Perturbing the initial steady state, which was also set in this case, had no influence on the stability, i.e. the system is resistant with respect to a change in initial steady-state concentrations (see the electronic supplementary material, figure S9). In figure 6, we plot the stability regions of the steady state in the two-dimensional 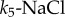 and 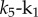 plane. For other parameter combinations, please refer to the electronic supplementary material, figure S9. Notably, the parameters of both the system with autoinhibition (model no. 6) and the system without autoinhibition (model no. 2) are rather similar (black and grey dots in figure 6 and the electronic supplementary material, S9). This indicates that the stability of a system can be modified by changing the system's structure by autoinhibition without significantly affecting other system parameters and, therefore, its dynamics (figure 5 and the electronic supplementary material, S8).

**Figure 7.**
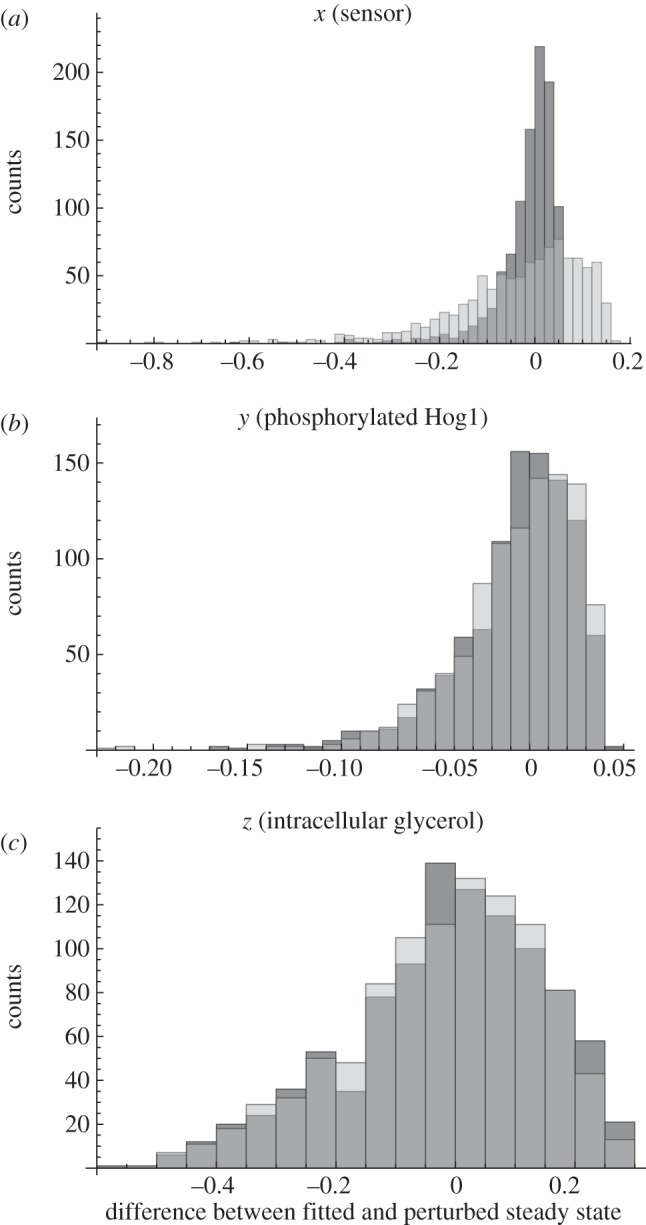
Monte Carlo analysis of the best approximating model with and without autoinhibitory feedback of the HOG system. (*a*–*c*) Shows the distributions of the difference between the original and the perturbed calculated steady states of the model with autoinhibitory feedback no. 6 (dark grey) and the model without autoinhibitory feedback no. 2 (light grey), respectively, for one of the three model components as indicated in the respective panel title. The dark grey regions indicate overlapping distributions. The free parameters (see electronic supplementary material, table S1) were perturbed by sampling 1000 times from a uniform distribution ranging from half to double of their original values and calculating the according steady states after an osmotic shock on 0.2 M NaCl.

It can be anticipated that in homeostatic adaptive systems the steady state should be robust against parameter perturbations. It has been shown that negative feedbacks can increase the robustness of the steady states with respect to input noise and parameter perturbations [4,11,12]. We hypothesized that nested autoinhibitory feedbacks can increase the robustness of the steady state. Therefore, we compared the steady-state variability with respect to parameter perturbation of the best model with feedback (model no. 6) and the best model without feedback (model no. 2) after an osmotic shock of 0.2 M NaCl in a Monte Carlo analysis. Specifically, we perturbed all free parameters of the system simultaneously by sampling 1000 times from a uniform distribution ranging from half to double of its original value. Subsequently, we calculated the distance between the original fitted steady state and the perturbed steady states (figure 7).

The variance of the distance between the original and the perturbed steady states of the sensor *x* is significantly smaller (*p* < 0.01) for the model with autoinhibitory feedback (model no. 6) compared with the model without autoinhibitory feedback (model no. 2; figure 7). In addition, the respective variance for components *y* is smaller (*p* < 0.01; the electronic supplementary material, table S1). For the response *z*, no significant difference was detected. Thus, in this concrete case, the autoinhibitory feedback increases robustness of the steady states after osmotic shock for the sensor and activated Hog1.

Taken together, our three-dimensional framework is able to recapitulate well a range of data for different conditions for the HOG system. Model discrimination suggests that in the HOG system there are autoinhibitory feedbacks nested within the glycerol-mediated feedback, and the system is stimulated by the difference between internal and external conditions. This is well supported by other studies [17,21]. Moreover, the autoinhibitory feedback renders the steady state of the system more resistant in the sense that parameter perturbations and external stress conditions are unlikely to drive the system beyond the threshold where oscillations occur.

### 2.7. Application to the p53 system

The p53 system is one of the best-studied human signalling pathways, which is activated by various stress signals, including DNA damage [40,41]. Interestingly, p53 phosphorylation can exhibit both oscillatory behaviour and sustained activation, depending on the stimulus, which imply different cell fates [26,42]. A range of models have been developed for this pathway to understand dynamics and variability of the protein circuitry [24,25,43]. Here, we tested whether our modelling framework can also explain p53 and Mdm2 dynamics, possibly giving new insights into the feedback regulation circuitry of the system. To this end, we fitted again different model alternatives based on our general framework (2.1) to a published average oscillation pattern of p53 and Mdm2 dynamics after DNA damage [25] (figure 4*b*):2.12
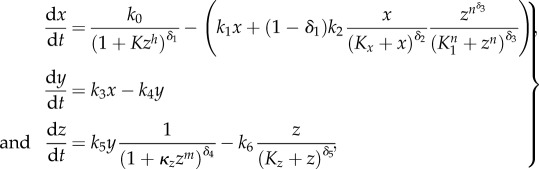
where *δ* = (*δ*_1_, *δ*_2_, *δ*_3_, *δ*_4_, *δ*_5_), 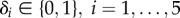, indicate model alternatives. Now, our model components are interpreted such that the signal *x* is p53 activation (sensing e.g. DNA damage), and the transducer *y* is an intermediate component, e.g. Mdm2 RNA. Consequently, for the latter component, no mass conservation is assumed, i.e. without the term (*y_t_* − *y*) in (2.1). The response *z* represents Mdm2 protein concentration which, in turn, mediates p53 degradation. Like for the HOG model, we tested two kinetic alternatives for reactions *f*_1_(*x*), and *h*(*z*), i.e. mass action (*δ*_2_, *δ*_5_ = 0) or Michaelis–Menten kinetics (*δ*_2_, *δ*_5_ = 1). In the p53 models, we assumed 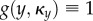, because, assuming the transducer to be RNA, a fast autoinhibitory feedback seemed unlikely. Therefore, we tested autoinhibition in component *z* by alternatively introducing 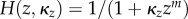 (*δ*_4_), assuming the fast autoinhibitory feedback to act at the protein level by, e.g. post-translational modifications. The kinetic nature of the negative feedback of Mdm2 on p53 remains elusive. Therefore, we also tested here two different alternatives for *f*_3_(*z*), i.e. mass action (*δ*_3_ = 0) and Hill-type kinetics (*δ*_3_ = 1). Additionally, we tested, as for the HOG system, the possibility that the negative feedback acts by input-inhibition (*δ*_1_ = 1) or by output-activation (*δ*_1_ = 0). Combination of the different possibilities results in 20 different models. The result of the fitting and ranking is displayed in table 2. For parameters of the best-ranked model, please refer to the electronic supplementary material, table S3. A COPASI implementation of the best-ranked model together with the data and an SBML version is also provided in the electronic supplementary material.

**Table 2.** p53 candidate models. *n*, number of data points, *k*, number of parameters; SSR, weighted sum of squared residuals; AIC*c*, Akaike information criterion corrected for small sample size; AIC*w*, Akaike weights.

rank	model	*δ*	*n*	*k*	SSR	AICc	AICw
1	11	(0,0,1,0,1)	91	10	1.8	−76.8	1.0
2	15	(0,0,1,1,1)	91	12	1.8	−68.8	0.02
3	16	(0,1,1,1,1)	91	13	2.8	−26.4	0
4	10	(0,1,1,0,0)	91	10	2.9	−31.1	0
5	14	(0,1,1,1,0)	91	12	3.0	−25.6	0
6	7	(0,0,0,1,1)	91	10	4.5	7.3	0
7	3	(0,0,0,0,1)	91	8	5.0	12.2	0
8	4	(0,1,0,0,1)	91	9	5.0	14.2	0
9	8	(0,1,0,1,1)	91	11	5.1	20.7	0
10	9	(0,0,1,0,0)	91	9	5.6	25.1	0
11	13	(0,0,1,1,0)	91	11	5.6	30.3	0
12	18	(1,0,0,0,0)	91	9	10.5	81.8	0
13	20	(1,0,0,1,1)	91	11	11.0	91.6	0
14	17	(1,0,0,0,0)	91	8	12.0	91.6	0
15	6	(0,1,0,1,0)	91	10	12.4	99.9	0
16	2	(0,1,0,0,0)	91	8	13.3	101.0	0
17	19	(1,0,0,1,0)	91	10	16.1	123.1	0
18	1	(0,0,0,0,0)	91	7	16.7	119.1	0
19	5	(0,0,0,1,0)	91	9	16.7	124.0	0
20	12	(0,1,1,0,1)	91	11	18.1	136.7	0

**Figure 8.**
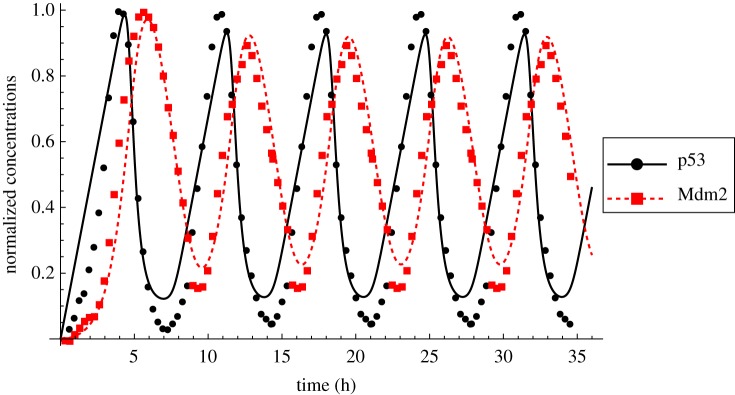
Data (adapted from [25]) and fit of the best approximating p53 model no. 11, *δ* = (0, 0, 1, 0, 1). Measured (symbols) and simulated (lines) concentrations for p53 (*x,* black, solid) and Mdm2 (*z,* red, dashed). For the source of the experimental data, please refer to section Materials and methods. (Online version in colour.)

The two best-ranked models (no. 11, *δ* = (0, 0, 1, 0, 1) and no. 15, *δ* = (0, 0, 1, 1, 1)) feature mass action kinetics in *f*_1_(*x*), Hill-type kinetics in *f*_3_(*z*), and zero-order kinetics in *h*(*z*) and their fit is significantly better than for the other model candidates. Whether or not these two models have an autoinhibitory feedback does not influence the goodness fit itself (sum of squared residual (SSR) in table 2), but as the model without autoinhibitory feedback has two parameters less, it is clearly ranked first. The fit of the best approximating model no. 11 is shown in figure 8.

**Figure 9.**
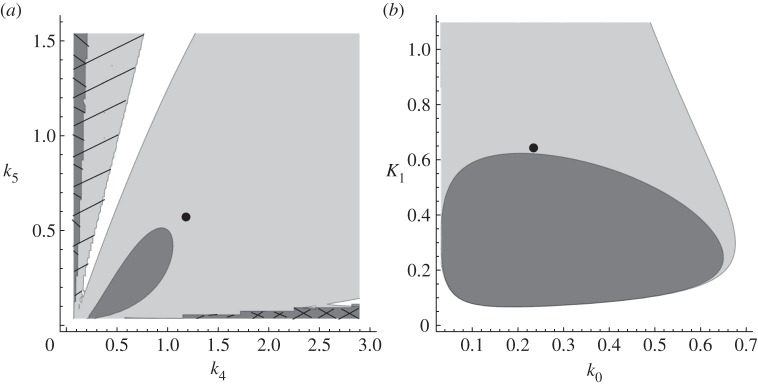
Stability region plots for the best approximating p53 model (model no. 11). (*a*,*b*) Shows stability regions for different parameter combinations. In (*a*,*b*), dark grey and light grey indicate regions with unstable steady state, i.e. where oscillations occur, for the system with a feedback of the form 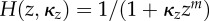 (*κ*_*z*_ = 1.95 and *m* = 3), and without autoinhibitory feedback, respectively. Black dots indicate the fitted parameters for the best approximating p53 model (model no. 11). The hatched regions indicate parameter combinations without steady state. The boundary between white and shaded regions indicates bifurcation lines in the respective two-dimensional parameter space. For more stability region plots for different parameter combinations, please refer to the electronic supplementary material.

The p53 system can show both oscillatory as well as sustained behaviour, depending on the stimulus [26,42]. Therefore, we asked the question whether the oscillations of the best approximating model can be stabilized by introducing a fast autoinhibitory feedback. This is only true in general, i.e. irrespective of the other parameters, when *f*_1_(*x*) has zero-order kinetics at the equilibrium, which is not the case for the best approximating p53 model no. 11. However, with the given set of parameters, our fitted model can indeed be stabilized by the introduction of an autoinhibitory feedback of the form 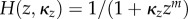, with *κ*_*z*_ = 1.95 and *m* = 3 (figure 9).

Figure 9 depicts the stability of the steady state of the best approximating model with and without autoinhibitory feedback as a function of selected parameters (for additional pairs of parameters, refer to the electronic supplementary material, figure S10). Like for the HOG model, the unstable region diminishes through the introduction of a nested autoinhibitory feedback. Moreover, the stability of the system changes upon addition of the nested autoinhibitory feedback, rendering the system in the stable zone after introduction of an autoinhibitory feedback.

**Figure 10.**
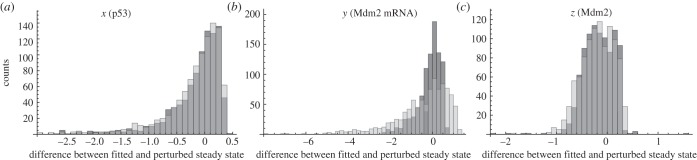
Monte Carlo analysis of the best approximating model with and without autoinhibitory feedback of the p53 system. (*a*–*c*) Shows the distributions of the difference between the original and the perturbed calculated steady states of the model with autoinhibitory feedback no. 15 (dark grey) and the model without autoinhibitory feedback no. 11 (light grey), respectively, for one of the three model components as indicated in the respective panel title. The dark grey regions indicate overlapping distributions. The free parameters (see electronic supplementary material, table S3) were perturbed by sampling 1000 times from a uniform distribution ranging from half to double of their original values and calculating the according steady states.

For the p53 system, a Monte Carlo analysis of the steady state with respect to parameter perturbations also indicated that the system with autoinhibitory feedback is less sensitive (figure 10).

The variance of the distance between the original and the perturbed steady states for all steady states is significantly smaller (*p* < 0.001) for the model with autoinhibitory feedback (model no. 15) compared with the model without autoinhibitory feedback (model no. 11; figure 10 and the electronic supplementary material, table S4).

Taken together, our simple framework suggests a mechanism how the p53 signalling system can change its dynamic behaviour upon different stimuli. Certain stimuli might activate components which introduce a nested autoinhibitory feedback. This changes the stability landscape of the system, shifting it from an oscillatory regime into a stable one. Thus, the p53 system depicts low resistance to parameter perturbations in order to be able to change its stability properties depending on environmental conditions.

## 3. Discussion

The ability to adapt to perturbations in external or internal conditions without losing structural stability is a fundamental feature of biological systems, including ecological, climate or biochemical systems. Adaptation is often mediated by negative feedbacks [1,7]. In biochemical systems, negative feedbacks inevitably come with time delays, which may lead to oscillatory behaviour both damped and sustained [15,23]. In some instances, oscillatory behaviour might oppose efficient adaptation owing to recurring stress. In such cases, the distance between the state of a system and the threshold beyond which oscillations occur, i.e. the systems resistance, should be large. This way, perturbations can be absorbed without affecting the structural stability of the system. In other instances, however, it might be beneficial to be able to switch between different dynamic regimes. It has been shown that the difference between a sustained or oscillatory signal can control cell fate [26,44], and that oscillation frequency can encode biochemical information [45]. In that case, resistance of a system should be low to be able to easily shift between different stability regimes. It might also be desirable to synthetically engineer cellular systems in a way such that oscillatory behaviour can be tuned by an independent artificial component.

For a three-dimensional system, it has been observed that coupling autoinhibitory and delayed negative feedbacks reduces the probability of occurrence of stable limit cycles [5]. For the simple gene transcription network model with input-inhibition proposed by Goodwin [15], it has been shown that nested self-repressing feedback loops have the potential to suppress oscillations [32]. Here, we propose a generic mechanism, how adaptive homeostatic biochemical systems can control both its dynamic response and its distance to a threshold beyond which these dynamics are drastically altered. We provide complete proof that in generic three-dimensional homeostatic adaptive biochemical networks both with input-inhibition as well as with output-activation oscillations may arise due to a Hopf bifurcation. We further prove that nested autoinhibitory feedbacks diminish the parameter space in which the steady state becomes unstable and oscillations occur. For systems with input-inhibition, the region for oscillations is generally reduced by autoinhibitory feedbacks. This is also true for models with a signalling module (mass conservation) and perfect adaptation (zero-order *h*(*z*)). The latter renders input-inhibition systems susceptible to oscillations also for low cooperativity in the input-inhibition which extends former studies [16,32,46]. For our system with output-activation, this is only true irrespective of the parameters, when the feedback-activated output is of zero-order. Thus, this is a sufficient, but not necessary condition. We show that this condition also has as the consequence that the system is stimulated by the positive difference between external and internal conditions. Our applications to the HOG and the p53 system suggest that zero-order output-activation might be a biologically more relevant feedback mechanism than input-inhibition. For the adaptive HOG system, we also demonstrate that a nested autoinhibitory feedback can alter the structural stability of the system without significantly affecting parameters of the system that are not involved in this feedback. Therefore, autoinhibition can alter stability properties of a system without affecting dynamic properties within a certain range of conditions. Owing to the generality of our model, we may hypothesize that other kinds of nested feedbacks also have the potential to suppress oscillatory behaviour. Here, we analysed only autoinhibitory feedback for mathematical convenience. However, in the special case of a Goodwin-type model, it has been shown that a nested feedback from *z* to *y* also diminishes the parameter region in which oscillations occur [32]. In addition, for a four-dimensional model, it has even been demonstrated that a feedforward loop within an integral negative feedback also diminishes the parameter regions in which oscillations occur [21].

The application of our system to the HOG and the p53 system also provides evidence that nested autoinhibitory feedbacks increase the robustness of the steady state with respect to parameter perturbations. For the ERK pathway, it has been observed that a fast post-translational feedback mechanism confers robustness to steady-state phosphorylation of ERK [47], which supports our analysis.

Our results may have implications to understand the complex dynamics of a range of signalling pathways. Not only has the p53 system been shown to exhibit different dynamics depending on the stimulus. The ERK pathway can show both oscillatory and adaptive dynamics, which are likely due to different feedback mechanisms that act on different timescales and that are activated depending on the stimulus [47–49]. The NF-κB system can show damped oscillations, which are likely due to different feedback mechanisms acting on different timescales [27]. It seems that the coupling of fast post-translational and delayed transcriptional feedbacks is a general feature of signalling pathways that allows fine-tuning of dynamics and steady-state features. The role of fast post-translational negative feedbacks in this respect is apparently either to suppress oscillatory behaviour or stabilize steady-state protein levels or both.

The presented theoretical results on suppressing oscillatory behaviour induced by Hopf bifurcations may be useful in designing synthetic systems in which oscillations can be tuned by synthetic autoinhibitory feedbacks. This may be useful for studying cell fate decisions, as, for example, in the p53 or the ERK system. For the HOG system, the parametrized models show that even without autoinhibitory feedback osmo-adaptation is extremely stable. For this system, it seems unlikely that oscillations can be induced artificially by weakening the reported autoinhibitory feedbacks.

## 4. Material and methods

### 4.1. Data

We made extensive use of published data to parametrize dynamic models of the HOG pathway and the p53 pathway. The dataset used for model parametrization and discrimination of the HOG model was taken from [17]. This dataset consists of time series of phosphorylated Hog1 under several hyper-osmotic shock conditions, for wild-type and different mutants yeast, for up to 2 h after hyper-osmotic shock (figure 5*a,b*). Additionally, we used a time series of glycerol published in [22] (figure 5*c*). These datasets, although coming from different sources, are comparable because they were produced using the same genetic background and under the same culture conditions. The dataset used for model parametrization and discrimination of the p53 model was digitized from the electronic supplementary material, figure S6 of the supplementary material of [25]. These data are meant to resemble an idealized undamped oscillation with peak characteristic that correspond to the average peak characteristic of oscillating cells. For the ranking procedure, we considered only 91 data points, because the last three periods in figure 8 are repetitions of the former oscillations.

### 4.2. Model fitting, ranking and selecting

The models were implemented and fitted with the free software COPASI (v. 4.7, build 34) [50]. We used the evolutionary programming algorithm to fit the models, where the population size was set to 10 times the number of parameters and the number of generations was limited to 10 times the number of parameters. When estimated parameters hit parameter boundaries, the boundaries were relaxed and the model refitted until the fit converged within defined parameter boundaries. Model ranking was performed using modelMaGe [51,52]. For model ranking, we calculated the Akaike information criterion corrected (AICc) for small sample sizes [53] for each candidate model:
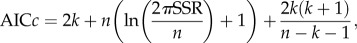
where SSR is the sum of squared residuals of the fit, *k* is the number of parameters and *n* is the number of data points. The AIC*c* is an information-theory-based measure of parsimonious data representation that incorporates the goodness of the fit (SSR) as well as the complexity of the model (*k*), thereby giving an objective measure for model selection and discrimination.

In order to select and compare the best approximating model(s), we calculated the Akaike weights (AIC*w*) [53]
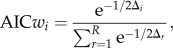
where *Δ_i_* = AIC*_i_* − AIC_min_, with AIC*_i_* being the AIC*c* for model *i*, *i* = 1,*…*, *R* according to ranking and AIC_min_ the minimal AIC*c*. The AIC*w*s can be considered as the weight of evidence in favour of a model given as a number between 0 and 1, i.e. the higher the weight, the closer the model is to the hypothetical true model [53]. We considered those models as best approximating that had an 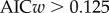.

## Funding statement

This study was supported by the German Ministry of Science and Education (BMBF project no. 0135779 and 0316188E to J.S.) and the International Max Planck Research School Magdeburg for Advanced Methods in Process and Systems Engineering. The authors declare no conflicting interests.
